# Plant Functional Traits and Soil Properties Shape Soil Microbial Communities in *Larix principis-rupprechtii* Mixed Plantations

**DOI:** 10.3390/biology15030259

**Published:** 2026-01-30

**Authors:** Zhaoxuan Ge, Bo Peng, Xiaotong Chen, Junfei Zhang, Ziyi Wang, Yue Pang, Zhidong Zhang

**Affiliations:** 1 College of Forestry, Hebei Agricultural University, Baoding 071000, China; 2 Forestry and Grassland Survey & Planning Institute of Hebei Province, Shijiazhuang 050090, China

**Keywords:** leaf traits, soil bacteria and fungi, co-occurrence network, keystone microbial taxa, tree species mixing, managed forest

## Abstract

The mixing of tree species influences soil microbial communities and ecosystem functioning, yet the underlying mechanisms remain inadequately understood. This study aimed to elucidate how different tree species mixtures regulate soil microbial community structure and ecological functions and to disentangle the relative roles of leaf functional traits, soil nutrients, and tree species identity in shaping bacterial and fungal assemblages. Leaf and soil samples were collected from 15 plots (20 m × 30 m) established in pure *Larix principis-rupprechtii* plantations (PL) and mixed *Larix*-*Betula platyphylla* (MLB) and *Larix*-*Picea asperata* (MLP) stands in the Saihanba Mechanical Forest Farm, China. Principal coordinate analysis, co-occurrence network analysis, and partial least squares path modeling were employed to assess changes in microbial community structure, network organization, and functional potential. Our results showed that the MLB stand mainly improved leaf nitrogen content (LNC), specific leaf area (SLA), and the concentrations of total nitrogen (STN) and phosphorus (STP) in the soil. The MLP stand preferentially promoted carbon and phosphorus accumulation in both leaves and soil. The MLB stand exhibited higher bacterial Chao1 richness, whereas the MLP stand showed reduced fungal diversity. The MLB supported a more complex bacterial network enriched with keystone taxa involved in nitrification and nitrate reduction, while MLP displayed a less complex bacterial network and a higher relative abundance of ectomycorrhizal fungi. Path analyses revealed that tree species mixtures shaped bacterial community structure largely via changes in leaf functional traits and soil conditions. Bacterial functional potential was primarily driven by improvements in soil nutrient availability. In contrast, fungal assemblage organization and functional expression were directly governed by the identity of the mixed tree species. These insights provide a foundation for improving soil fertility and nutrient cycling in managed forests via strategic species diversification.

## Simple Summary

It is vital to understand how mixing different tree species can make forests healthier, but the specific ways this works underground are not fully clear. In this study, we compared the soil microbial community from pure larch forests with mixed forests where larch grows together with birch or spruce. We collected leaves and soil to understand how different tree combinations influence soil microorganisms. We found that mixing larch with birch increased leaf quality and soil nutrients, such as nitrogen and phosphorus, and supported a richer and more interactive community of soil bacteria that help transform nutrients. Mixing larch with spruce mainly increased carbon and phosphorus storage but supported fewer soil fungi, especially favoring fungi that form close partnerships with tree roots. Our results show that bacteria are influenced indirectly by changes in leaf traits and soil nutrients, while fungi are more directly shaped by the type of tree present. These findings help explain how tree diversity can be used to improve soil fertility, nutrient cycling, and the long-term sustainability of managed forests.

## 1. Introduction

Artificial forests, a key component of forest ecosystems, are critical for improving soil quality and regulating ecosystem carbon cycling [1,2]. However, most afforestation has been established as monocultures [3], leading to reduced ecological stability and productivity [4,5]. In contrast, mixed-species plantations are generally considered an effective afforestation strategy to mitigate the ecological drawbacks posed by monoculture systems [4,6]. These ecological benefits stem not only from the specific composition and interspecific interactions in the plant community [7,8] but also from the complex belowground processes that mixed stands promote [9]. The microbial communities in the soil are crucial in forest ecosystems, as they drive essential biogeochemical mechanisms responsible for regulating ecosystem material flows, such as the decomposition of plant litter, organic matter turnover, and the cycling of vital nutrients, all of which are fundamental to maintaining forest productivity and ecological stability [10,11,12]. Nonetheless, the structure and functioning of soil microbial communities are profoundly influenced by a diverse array of environmental drivers, such as soil chemical and physical conditions, climate variation, and attributes of the overstory vegetation [13,14]. Consequently, identifying the primary drivers of microbial community structure and their functional potential has become a critical objective in forest ecology, providing essential insights for predicting and managing ecosystem services.

Tree species mixtures form more complex systems than their corresponding monocultures. In these systems, different plant species and the communities they assemble have been shown to regulate soil microbial composition and modulate key ecosystem processes [15,16] by providing diverse litter input, root symbionts, and microhabitat conditions [17,18]. According to Bai, Wei, Ming, Shu, and Shen [12], tree species mixing increases soil bacterial diversity and alters community composition, which is often linked to improvements in soil factors, such as changes in soil pH [19], elevated organic matter content [20], and improved nutrient cycling [21]. Conversely, the diversity and composition of the fungal community appear to be more directly driven by the functional identity of the vegetation [20,22]. Consequences of tree species mixtures for soil characteristics and their associated microbial communities are primarily driven by complementary plant traits, especially leaf functional traits [23]. Leaves, being highly responsive to environmental changes [24,25], possess functional traits that persist after abscission and serve as key indicators linking plant adaptive strategies to ecosystem functions [26]. These traits largely determine both the resource input and the quality, as returned to the soil through litterfall [27,28]. Prior research has shown that litter inputs constitute a primary pathway through which plants influence soil microbial communities, as litter provides multiple essential nutrients and energy-rich substrates required for microbial growth [29,30,31]. Freschet et al. [32] further showed that litter produced by species with acquisitive strategies—typically marked by higher nutrient levels and lower lignin concentration—decomposes more rapidly and supports greater microbial activity. Consequently, plant functional traits can influence soil microbial assemblages in multiple ways, exerting direct resource inputs while also generating indirect impacts mediated by changes in soil characteristics and microhabitat conditions [33].

Despite these insights, the mechanisms through which leaf functional traits regulate bacterial and fungal communities remain insufficiently explored. Moreover, the influence of plant attributes on belowground ecological processes cannot be fully elucidated solely by analyzing changes in microbial composition and diversity. Compared with the numerous non-dominant microbial groups, those considered ecological keystones exert an outsized influence on sustaining ecosystem functioning [34]. These taxa serve as central nodes in microbial co-occurrence networks and are essential for maintaining community stability and performing specialized ecological functions [35]. Therefore, functional prediction of these keystone taxa provides valuable insights into their genetic and metabolic potential, enhancing our understanding of how microbial processes in mixed forests contribute to critical ecosystem functions, particularly the cycling of essential soil nutrients.

The coniferous tree larch (*Larix principis-rupprechtii*) dominates the forests of northern Hebei Province, China. Although larch plantations contribute substantially to timber production, ecological restoration, and the maintenance of regional carbon and water cycles [36,37], their extensive monoculture expansion has resulted in declining productivity, reduced soil fertility, and diminished ecosystem stability [38,39]. Our previous work in this region has shown that greater diversity among surrounding tree species can lead to higher stand productivity than that observed in pure larch plantations [40]. Furthermore, mixed-litter treatments have been shown to promote faster litter decomposition and improve soil physicochemical properties [39]. These findings indicate that tree species mixing can improve the stand microenvironment, while changes in plant resource-use strategies reflected by variation in leaf functional traits may subsequently alter soil microbial community composition. Nevertheless, how shifts in leaf functional attributes and soil environmental conditions induced by tree species mixing alter microbial assemblages in the soil remains insufficiently understood.

To evaluate the influence of larch mixed plantations on soil microbial communities and their associations with leaf functional traits and soil properties, we analyzed changes in microbial composition, diversity, and keystone taxa functions across pure larch plantations (PL), and mixed larch-*Betula platyphylla* (MLB) and larch-*Picea asperata* (MLP) stands in the Saihanba area, China. In these stands, broadleaved species such as birch (*B*. *platyphylla*) generally produce higher-quality litter with lower C/N ratios and fewer recalcitrant compounds than the evergreen coniferous spruce (*P*. *asperata*) [41]. These contrasts in litter chemistry tend to accelerate decomposition rates and soil nutrient mineralization, thereby contributing to the observed differences in microbial functional profiles among stand types [42]. The study pursued three main goals: (1) to clarify how different admixed tree species drive soil microbial assemblages, including shifts in their diversity, community composition, and the ecological roles of keystone taxa; (2) to assess the contributions of species identity, leaf functional traits, and species-mediated soil properties in influencing microbial communities; and (3) to elucidate the potential mechanisms through which tree species admixture affects microbial ecological functions. Overall, this study seeks to advance the understanding of plant-soil feedback in mixed stands, providing a scientific basis for optimizing tree species selection and enhancing forest ecosystem services.

## 2. Materials and Methods

### 2.1. Study Site and Experimental Design

**Figure 1:**
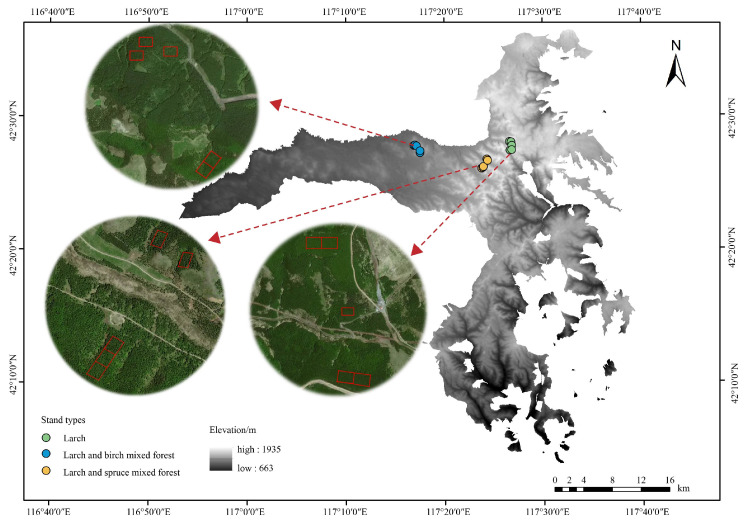
Sampling plot locations in Saihanba Mechanical Forest Farm, northern Hebei Province, China.

The investigation was carried out in the Saihanba Mechanical Forest Farm (42°02′–42°31′ N, 116°53′–117°39′ E; Figure 1), located in Hebei Province, China, covering an area of approximately 93,333 km^2^. The region has achieved a forest coverage of 82% with plantations occupying about 76, 700 km^2^. As a region of continental monsoon climate, the area exhibits long winters that result in a sub-zero mean annual temperature. Precipitation is moderate (450–500 mm) and predominantly occurs in summer, while the region has a brief frost-free period of approximately two months and marked diurnal temperature variation. The elevation ranges from 1100 m to 1940 m, with a terrain gradient that decreases from north to south [37]. The soil of the region falls within the category of gray forest soils according to China’s national soil classification, which aligns with Mollisols in the USDA Soil Taxonomy framework. In the early 20th century, extensive overgrazing and intensive logging led to severe degradation of the region’s natural forests. To restore the damaged ecosystem, large-scale afforestation with larch and other cold- and drought-tolerant species, such as spruce, was initiated in 1962. The MLB and MLP stands developed naturally following the initial plantation establishment through regeneration from adjacent secondary forests. At present, forest coverage in the region has reached 82%, with plantation forests occupying approximately 76,700 km^2^, making it one of the largest artificial forest farms in the world. All studied stands share a comparable silvicultural history, characterized primarily by thinning for stand structure regulation and the absence of fertilization or other intensive management practices. Larch, spruce, birch, and *Pinus sylvestris* var. *mongolica* are the characteristic dominant canopy species. The main shrub and herbaceous species under the forest include *Malus baccata*, *Rosa davurica*, *Sanguisorba officinalis*, and *Agrimonia pilosa*.

In July 2020, three representative forest types with comparable initial site conditions and stand age were selected: PL, MLB, and MLP. All sampling plots were deliberately established on flat terrain with minimal microtopographic variation to minimize the potential effects of slope position and aspect. According to tree ring studies, the stands were between 45 and 50 years old. To facilitate leaf and soil sample collection, a total of 15 permanent sampling plots were established, with five plots per forest type, each covering an area of 20 m × 30 m (Figure 1). A comprehensive inventory was conducted, documenting all standing trees with a diameter at breast height (DBH) of at least 5 cm. For each tree, measurements of height, DBH, and crown diameter were recorded. The basic stand characteristics are summarized in Table S1.

### 2.2. Leaf Sampling and Trait Determination

This research quantified six key leaf functional traits across the different larch forest types: LA, SLA, leaf dry matter content (LDMC), and the concentrations of leaf carbon (LCC), nitrogen (LNC), and phosphorus (LPC). In each sampling plot, five representative individual trees of each species (larch, birch, and spruce) were chosen at random for trait measurements. For each sampled tree, at least five mature, healthy, and pest-free leaves were obtained from the middle canopy using a combination of climbing techniques and pole pruners. The collected leaves were then immediately placed in paper bags and promptly delivered to the laboratory for subsequent processing. LA was determined as projected leaf area by scanning leaves at 300 dpi resolution in grayscale mode, after which the images were analyzed using ImageJ software (version 1.43u). Prior to scanning, leaves were carefully arranged flat on the scanner surface to avoid overlap. The needles were manually separated and evenly distributed in a single layer to minimize measurement bias. Fresh weight was first measured using a high-precision analytical balance (0.001 g), after which they were dried at 65 °C for no less than 48 h until reaching a constant mass. Dry weight was then recorded, and LDMC was determined as dry mass relative to fresh mass, while SLA was quantified as LA normalized by dry mass. LCC was determined using the potassium dichromate oxidation external heating method. LNC was measured using the Kjeldahl digestion method, and LPC was determined using the molybdenum–antimony colorimetric method. Leaf functional traits were calculated as community-weighted means (CWMs) [43].

### 2.3. Soil Sampling and DNA Sequencing

Soil samples were collected in July 2020. After surface litter was removed, five soil cores were taken at random from a depth of 0–20 cm in each plot with a 5 cm diameter coring device and formed into one composite sample. Altogether, 15 soil samples were obtained across the three stand types (3 stand types × 5 replicates). Each composite soil sample was subjected to sieving through a 2 mm mesh and then split into two subsamples. One subset was air-dried in the laboratory and used for physicochemical analysis. Soil chemical indicators, including pH, STN, STP, organic carbon (SOC), and the concentrations of available N (SAN) and P (SAP), were quantified following the standard agrochemical procedures outlined by Bao [44]. The remaining subsamples were loaded into disinfected 10 mL centrifuge tubes, rapidly snap-frozen in liquid nitrogen, and stored at −80 °C until genomic DNA isolation.

The extraction of soil genomic DNA from each sample was carried out with the OMEGA Soil DNA Kit (Omega Biotek, Norcross, GA, USA). Subsequent amplification of bacterial 16S rRNA fragments employed the 338F/806R primer set, while fungal ITS sequences were obtained using the ITS5 and ITS2 primer pair. The resulting PCR products were isolated with a gel purification kit from Axygen (Union City, CA, USA) and quantified fluorometrically using the FLx800 microplate reader (BioTek Instruments, Union City, CA, USA). Sequencing was carried out in paired-end mode on the NovaSeq platform from Illumina (San Diego, CA, USA). The raw reads were subsequently subjected to quality control and denoising through the DADA2 workflow implemented in QIIME2 (v2019.4), including sequence merging and chimera elimination to obtain high-resolution amplicon sequence variants (ASVs). Taxonomic classification of ASVs was performed with the Vsearch feature classifier using the SILVA (v132; bacteria) and UNITE (v8.0; fungi) databases.

The construction of microbial co-occurrence networks was conducted to elucidate stand type effects on microbial associations and to pinpoint keystone taxa, employing a conservative filtering approach that retained only bacterial and fungal ASVs (≥20% prevalence across the five replicate samples within each forest type or >0.01% relative abundance) to reduce false positives [45]. Similar approaches have been adopted in previous studies to construct robust microbial networks by retaining dominant or consistently occurring taxa. These studies often apply different numerical thresholds for bacteria and fungi to account for differences in dataset size and complexity while maintaining comparable levels of filtering stringency [46]. Co-occurrence networks were generated based on Spearman correlation matrices calculated using the “Hmisc” package [47]. Only correlations with an absolute coefficient of |r| ≥ 0.7 and adjusted *p*-values passing the Benjamini–Hochberg correction threshold (FDR < 0.01) were included when assembling the co-occurrence network [48]. A set of topological parameters of the networks (nodes, edges, diameter, density, mean degree, and mean path length) was quantified via the “igraph” R package (version 2.2.1) [49]. Network structures were then rendered in Gephi (v.0.10.1) with an undirected layout that utilized the Fruchterman–Reingold force-directed algorithm. For each node, the within-module (Zi) and among-module (Pi) connectivity values were obtained to assess its structural contribution and functional role [3]. Keystone taxa were recognized according to their topological roles. Nodes showing high within-module connectivity (Zi ≥ 2.5) but limited among-module links (Pi < 0.62) were identified as module hubs, whereas those exhibiting strong cross-module associations (Pi ≥ 0.62) but low Zi values served as connectors. Nodes that displayed both high Zi and high Pi values were defined as network hubs.

### 2.4. Data Analysis

We conducted all data processing and statistical analyses in R version 4.5.1 [50]. Differences among stand types in leaf functional traits, soil characteristics, and microbial richness and diversity indices (Chao1 and Shannon) were tested via one-way ANOVA followed by Tukey’s HSD for post hoc comparisons. Prior to these tests, assumptions of normal distribution and variance homogeneity were examined using the Shapiro–Wilk and Levene tests, respectively. To reduce dimensionality and mitigate multicollinearity among measured variables, principal component analysis (PCA) was applied independently to the leaf trait variables and soil physicochemical properties (Figure S1). The resulting PC scores were further included as explanatory variables in a partial least squares-based path model (PLS-PM). Principal coordinate analysis (PCoA) was employed to visualize the dissimilarity in microbial community dissimilarity based on Bray–Curtis distance. This was followed by a permutational multivariate analysis of variance (PERMANOVA) with the adonis function to evaluate the influence of stand type on community structure. To examine significant (*p* < 0.05) associations of plant traits and soil characteristics with microbial community structure, we applied the “envfit” function [51]. To evaluate the potential influence of plant functional traits and soil properties on microbial community composition, multiple linear regression models were constructed using the first principal coordinate (PCo1) of bacterial and fungal communities (derived from Bray–Curtis dissimilarity matrices) as the response variable, with the first principal component scores of leaf traits (Leaf PC1) and soil properties (Soil PC1) included as continuous explanatory variables. Variation partitioning through distance-based redundancy analysis (db-RDA) was used to assess how leaf traits and soil properties contribute to microbial community variation. Significant predictors were identified through forward selection with 999 permutations using the “ordiR2step” function, retaining only variables with significant effects (*p* < 0.05) and VIF < 5. The ecological roles of key bacterial and fungal taxa were referencing the FAPROTAX and FUNGuid databases, which are widely used tools for predicting microbial ecological roles based on their taxonomic classification. STAMP was used to evaluate functional differences among stand types [52], followed by Spearman correlation analysis to investigate relationships between key microbial functional abundance, plant leaf traits, and soil physicochemical variables. Multiple regression analysis was employed to quantify how differences in leaf functional traits and soil physicochemical factors influenced key functional responses of microbiomes (bacteria and fungi). This was followed by variance decomposition, using the “relaimpo” package, to assess their relative importance.

PLS-PM was employed to test the hypothesized causal pathways linking mixture tree species, leaf functional traits, soil physicochemical factors, and the structure and functional roles of microbial communities, including keystone microbial taxa. To capture distinct microbial patterns, separate PLS-PM models for bacterial and fungal datasets. The two mixed forest types were included as exogenous variables influencing microbial community dynamics, with both direct and indirect effects mediated by the first principal component of leaf traits (Leaf PC1) and soil properties (Soil PC1), for which a factor loading threshold of 0.7 was applied.

## 3. Results

### 3.1. Leaf Functional Traits and Soil Properties Across Different Stand Types

**Figure 2:**
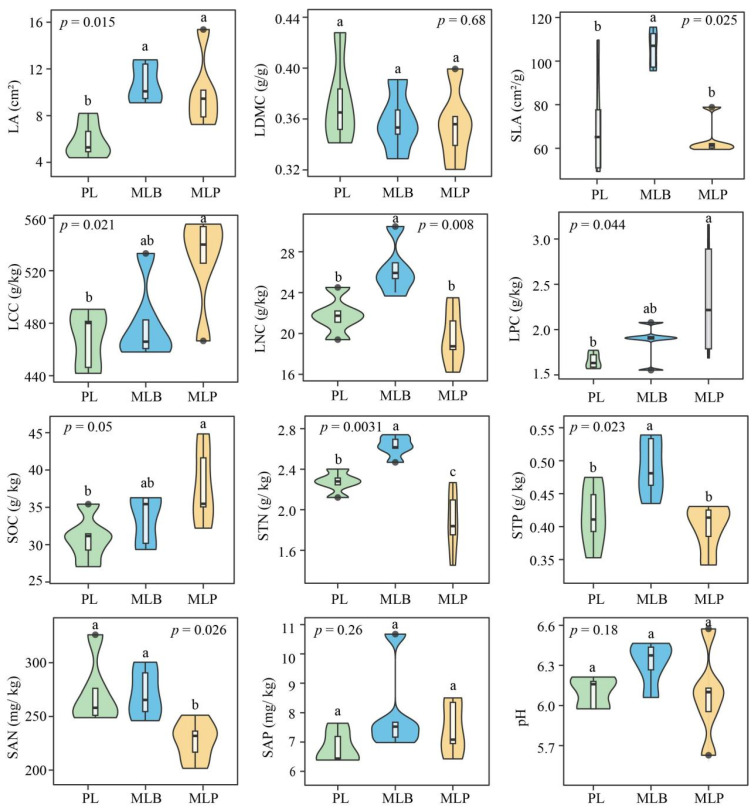
Differences in leaf trait variables and soil physicochemical properties were observed among stands. Distinct lowercase letters denote groups that are statistically different based on Tukey’s HSD test at the *p* < 0.05 threshold.

Among all leaf functional traits, all but LDMC varied across the three stand types (*p* < 0.05; Figure 2), indicating significant shifts in plant resource-use strategies associated with tree species composition. Notably, LA was markedly elevated in the two mixed-stand conditions relative to the PL stand (*p* = 0.0017), increasing by 72.62% and 55.75% in MLB and MLP stands, respectively. In addition, SLA and LNC were significantly higher in MLB stands (*p* < 0.01), reflecting a more resource-acquisitive leaf trait syndrome. In contrast, LPC and LCC reached higher values in MLP stands than in PL (*p* < 0.05), suggesting a comparatively more conservative investment in structural and nutrient traits. PCA of the leaf functional traits showed that Leaf PC1 explained 46.5% of the total variance and represented a resource economics spectrum, characterized by positive loadings of LNC and SLA but negative loadings on LCC and LPC. Leaf PC2, the second ordination axis, explained an additional 31.4% of the variance. Along this axis, positive scores corresponded to larger LA, whereas negative scores indicated a resource-conservative strategy associated with higher LDMC (Figure S1).

For soil physicochemical properties, univariate analysis indicated that MLB stands exhibited significantly higher STN and STP contents than PL stands (*p* < 0.05), consistent with the nutrient-enriched leaf trait profile observed in this mixed stand. Conversely, MLP stands showed significantly higher SOC but significantly lower SAN and STN content compared with PL stands (*p* < 0.05; Figure 2), indicating shifts in soil nutrient availability and carbon storage patterns associated with spruce admixture. SAP and pH did not exhibit significant variation across the stand types (*p* > 0.05). Analysis of soil properties through PCA revealed that the primary component, Soil PC1, captured 44.1% of the variance observed and primarily represented soil nutrient availability, with strong positive loadings on STN, STP, and SAN. Capturing 31.6% of the variance, Soil PC2 reflected a gradient of organic carbon and alkalinity, characterized by positive loadings on SOC and negative loadings on pH (Figure S1).

### 3.2. Response of Soil Microbial Community Diversity and Composition

**Figure 3:**
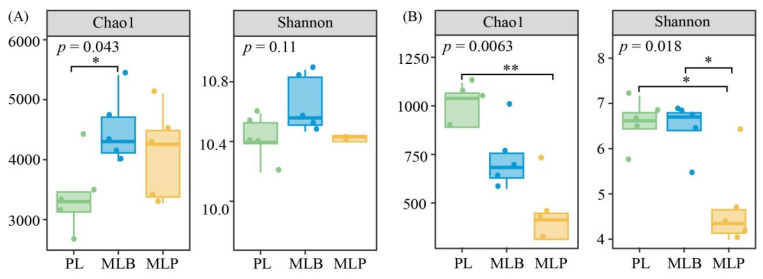
Bacterial (**A**) and fungal (**B**) α-diversity across larch stand types. Asterisks represent statistical significance (* *p* < 0.05; ** *p* < 0.01).

Regarding soil α-diversity, a significant increase in the bacterial Chao1 richness index was observed in MLB stands relative to PL stands (*p* < 0.05; Figure 3A). Differing from bacterial richness, no marked variation in the Shannon diversity index was detected among stands (*p* > 0.05; Figure 3A). Turning to fungal communities, both the Chao1 and Shannon indices exhibited significant reductions in MLP stands compared with PL and MLB stands (*p* < 0.05; Figure 3B). This indicated that spruce admixture exerted a stronger filtering effect on fungal community diversity.

**Figure 4:**
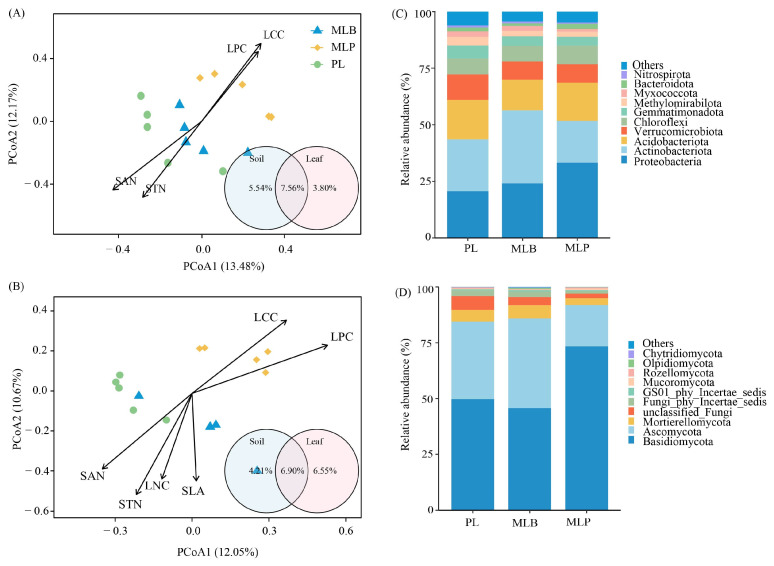
PCoA ordination of bacteria (**A**) and fungi (**B**), and phylum-level distribution of dominant taxa (**C**,**D**), with fitted vectors indicating significant factors (*p* < 0.05, Table S2). The inserted Venn diagrams illustrate the partitioning of microbial composition variance, highlighting the variance fractions uniquely attributed to leaf functional traits (pink circle) and soil properties (blue circle), as well as their shared contribution. Reported values are based on adjusted *R*^2^.

The PCoA ordination visualized distinct clustering patterns of microbial communities across the three stand types (Figure 4A,B). PERMANOVA revealed that stand type had a significant impact on the communities of both bacteria and fungi (*p* < 0.05; Table 1). For bacterial communities, the first two PCoA axes contributed to the total variance, explaining 13.48% and 12.17%, respectively (Figure 4A), while for fungal communities, these axes accounted for 12.05% and 10.67% of the variance (Figure 4B). Actinobacteria, Proteobacteria, Acidobacteria, and Verrucomicrobiota were the most prevalent bacterial phyla (Figure 4C), while Basidiomycota, Ascomycota, and Mortierellomycota were the predominant fungal phyla (Figure 4D). The composition of dominant microbial taxa varied substantially between bacteria and fungi. In the bacterial community, the five most abundant genera were *Uncultured*, *Candidatus_Udaeobacter*, KD4-96, *Xanthobacteraceae*, and *Rokubacteriales*, which collectively accounted for 42.99% of all bacterial sequences (Figure S2A). In contrast, the fungal community was dominated by *Russula*, *Sebacina*, *unclassified_Fungi*, *Leptodontidium*, and *Oidiodendron*, together comprising 37.84% of the total fungal sequences (Figure S2B).

Variation in bacterial and fungal community structures was significantly associated with leaf traits (LCC and LPC) and soil properties (STN and SAN) (envfit *p* < 0.05; Figure 4A,B, Table 2). Notably, the fungal community was more strongly associated with SLA (*R*^2^ = 0.38, *p* = 0.049) and LNC (*R*^2^ = 0.58, *p* = 0.006) (Table 2). Multiple regression analyses indicated that bacterial community variation was significantly predicted by soil properties (Soil PC1; *p* = 0.014), but not by leaf traits (Leaf PC1; *p* = 0.297). In contrast, neither soil properties nor leaf traits could significantly predict the variation in fungal communities (overall model *R*^2^ = 0.315, *p* > 0.05), although Leaf PC1 exhibited a marginal effect (*p* = 0.047) (Table S3), suggesting a tighter coupling between fungal communities and host plant functional strategies. The variation in bacterial communities was explained 16.90% by leaf functional traits and soil physicochemical properties, while these same factors accounted for 17.46% of the variation in fungal communities. Variation partitioning analysis further indicated that leaf traits alone accounted for 5.54% of the variation in bacterial communities, whereas soil factors independently explained 3.80% (Figure 4A). A similar pattern was observed for the fungal community, with leaf traits explaining 6.55% of the variation, exceeding the 4.21% explained solely by soil factors (Figure 4B).

### 3.3. Soil Microbial Co-Occurring Network and Functional Prediction

**Figure 5:**
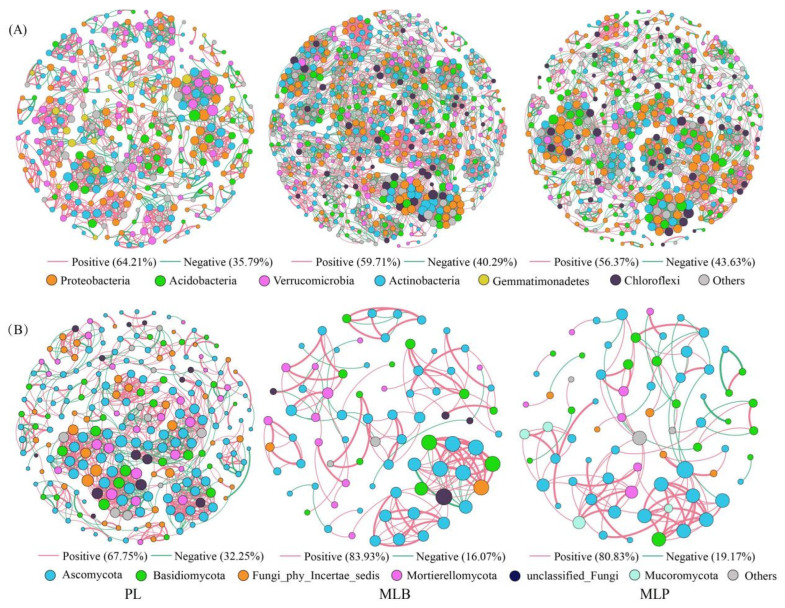
Visualization of phylum-level microbial co-occurrence networks for bacteria (**A**) and fungi (**B**) under three stands. The size of the node reflects its connectivity, with distinct colors marking individual phyla. Red lines indicate positive relationships, while green lines denote negative ones.

Microbial network complexity, as quantified by topological parameters, varied significantly among stand types (Figure 5). Compared with the PL stand, the bacterial networks in the MLB and MLP stands had more nodes and edge numbers and higher average degrees. In contrast, the MLB network showed a shorter average path length and smaller network diameter than the MLP network (Table S4). Bacterial networks in MLB and MLP stands contained more negative interactions than the PL network, and their overall topological complexity showed a gradually decreasing trend from MLB to MLP and then to PL (Figure 5A), indicating that broadleaf admixture promoted the most structurally complex bacterial networks. In contrast, the fungal networks in the PL stand (240 nodes and 1017 edges) exhibited higher complexity and a larger fraction of negative edges than those in the two mixed stands. Additionally, the MLB fungal network exhibited higher modularity and an average clustering coefficient (0.795 vs. 0.766 and 0.819 vs. 0.671, respectively) compared with the MLP network (Figure 5B; Table S4). Overall, these contrasting patterns indicate that bacteria and fungi responded differently to changes in tree species composition and associated environmental conditions.

Analysis of microbial co-occurrence networks indicated that bacterial communities contained a richer assemblage of keystone taxa than fungal communities (Table S5). In bacterial networks, the MLP and MLB stands harbored 30 and 27 keystone taxa, respectively, whereas the PL stand contained 19. A higher count of fungal keystone taxa was observed in the PL stand, followed by the MLP and MLB stands. The keystone bacterial taxa primarily belonged to Acidobacteria, Proteobacteria, and Actinobacteria, whereas the fungal networks were dominated by Ascomycota and Mortierellomycota (Figure 5). Moreover, most keystone taxa were categorized as connectors or module hubs, indicating their critical roles in maintaining network structure.

**Figure 6:**
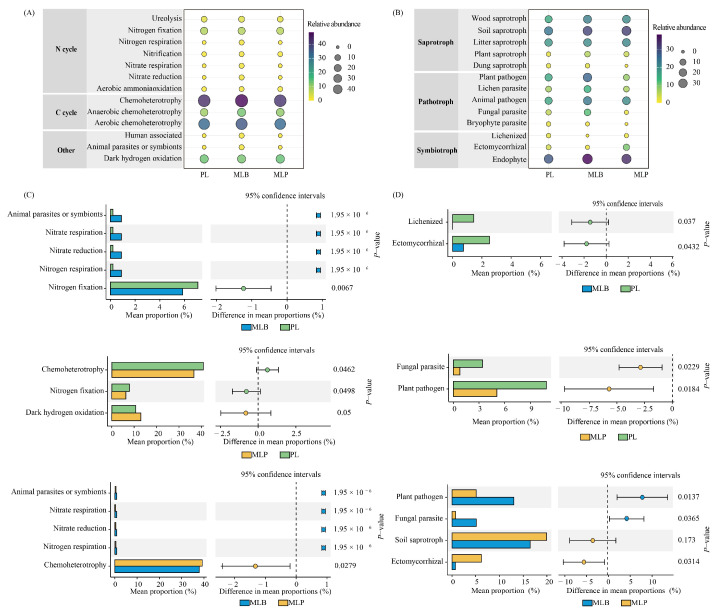
Composition of keystone ecological functions for bacteria (**A**) and fungi (**B**) in the three stand types. Keystone taxa for bacteria (**C**) and fungi (**D**) exhibited significant differences among stand types.

Functional prediction of keystone taxa revealed significant differences in ecological roles among the different forest stands (Figure 6). Bacterial keystone taxa participating in C and N cycling showed distinct abundance patterns, with both mixed stands exhibiting higher proportions than the pure PL stand (Figure 6A). Specifically, taxa associated with nitrification, nitrate reduction, and respiration occurred at significantly higher levels in the MLB stand compared with the PL and MLP stands (*p* < 0.05; Figure 6C). Conversely, taxa related to chemoheterotrophy and nitrogen fixation were more abundant in the PL stands, while those associated with dark hydrogen oxidation were more prevalent in the MLP stands than in the PL stands. The keystone fungal functional guilds were primarily dominated by saprotrophs and symbiotrophs, which occurred at higher relative abundances than pathotrophs (Figure 6B). The PL stand was significantly enriched in lichenized and ectomycorrhizal fungi compared with the MLB stand, whereas the latter exhibited the highest relative abundances of plant pathogens and fungal parasites (*p* < 0.05; Figure 6D). In addition, ectomycorrhizal fungi were more prevalent in the MLP stand than in the MLB stand, indicating differential fungal functional strategies across mixed-stand types.

**Figure 7:**
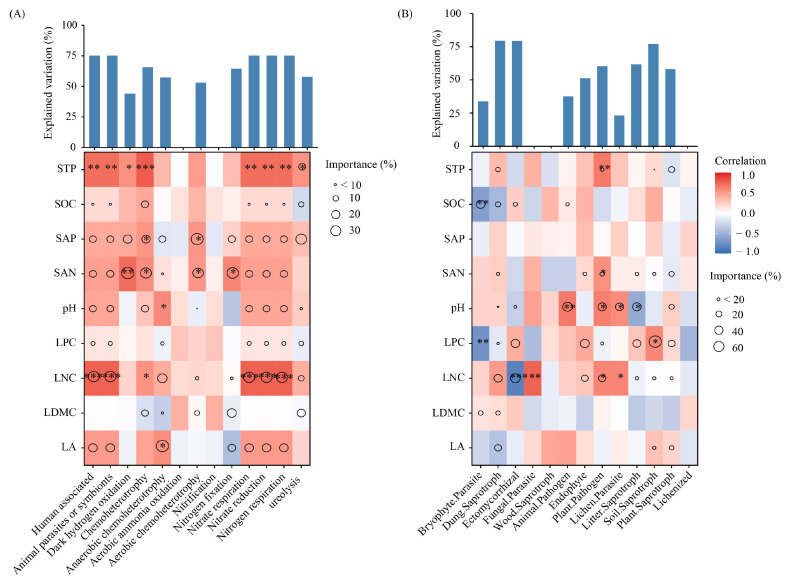
Contributions of leaf traits and soil properties to microbial functions for bacteria (**A**) and fungi (**B**), determined through correlation and optimal regression modeling. Circle size indicates its contribution to explaining variation in microbial functions, as inferred from optimal multiple regression and subsequent variance partitioning. Color intensity reflects Spearman correlation strength, and asterisks indicate significance levels (* *p* < 0.05; ** *p* < 0.01; *** *p* < 0.001).

Compared with keystone fungal taxa, bacterial keystone groups showed significantly stronger associations with soil properties (Figure 7), and their ecological functions were primarily influenced by STP, SAP, SAN, and LNC concentrations, together with pH and LA (*p* < 0.05, Figure 7A). On the other hand, the concentrations of STP, SOC, SAN, LPC, and LNC, along with pH, were identified as the dominant factors shaping the ecological functions of keystone fungal taxa (Figure 7B). STP and LNC were positively associated with the abundance of functional microbial groups involved in human association, animal parasitism or symbiosis, chemoheterotrophy, nitrate respiration, nitrate reduction, and nitrogen respiration. Both SAN and SAP were significantly related to the abundance of chemoheterotrophic and aerobic chemoheterotrophic taxa. Regarding fungal ecological functions, STP and SAN were positively associated with plant pathogens. Soil pH exhibited a significant positive correlation with animal pathogens, plant pathogens, and lichen parasites, but it was negatively correlated with litter saprotrophs. SOC and LPC exhibited significant negative correlations with bryophyte parasites. LNC was positively correlated with fungal parasites, plant pathogens, and lichen parasites, but it was negatively correlated with ectomycorrhizal fungi.

### 3.4. The Direct and Indirect Impacts on Bacterial and Fungal Structure and Function

**Figure 8:**
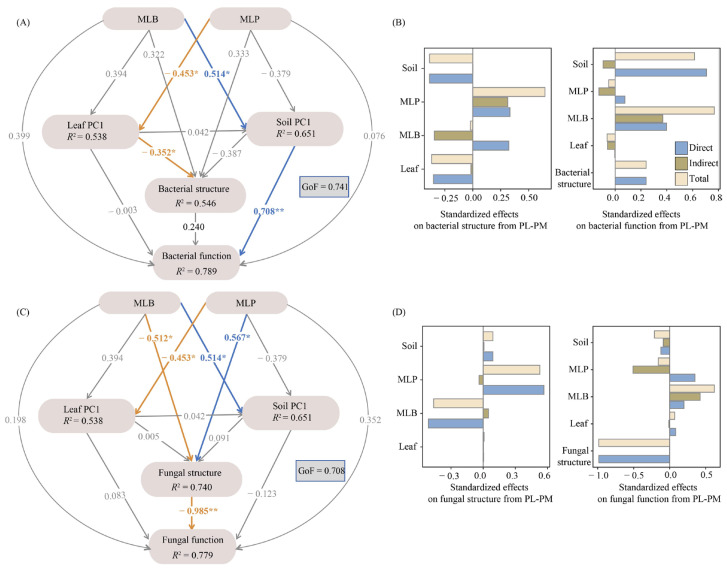
PLS-PM path diagram illustrating the influence of stand type (MLB, MLP), leaf functional traits (Leaf PC1), and soil physicochemical properties (Soil PC1) on both the structure and functional potential of bacteria (**A**,**B**) and fungi (**C**,**D**). Path coefficients (β) and explained variance (*R*^2^), derived from 999 bootstraps, are shown. Blue paths denote significant positive relationships, whereas yellow paths represent significant negative relationships (* *p* < 0.05, ** *p* < 0.01). Stand type was binary-coded (MLB = 1, MLP = 0). Overall model validity was quantified through the goodness-of-fit (GoF) metric.

The PLS-PM performed well in modeling both bacterial and fungal variables in the soil (GoF = 0.741 and 0.708, respectively), effectively capturing the primary source of variation in microbial community composition and its functional roles (Figure 8). Tree species mixture exerted significant direct effects on both leaf functional characteristics (Leaf PC1) and soil properties (Soil PC1); however, the regulatory patterns differed between the MLB and MLP stands. Tree species mixtures altered leaf trait patterns and soil conditions, which subsequently exerted a direct influence on bacterial community structure (Figure 8A). Notably, variation in leaf traits showed a significant direct negative association with bacterial community structure (β = −0.352, *p* < 0.05), suggesting that shifts along the leaf resource economics spectrum constrained bacterial compositional turnover. Although no statistically significant direct impact of soil properties was detected, their relatively high path coefficient (β = −0.387) suggests a potentially important role. At the functional level, keystone bacterial taxa were strongly shaped by stand type identity. The MLB stand was the primary driver of keystone bacterial function, with a total effect of 0.768 (Figure 8B). This effect comprised both a significant direct component and an indirect component mediated by improved soil properties. Conversely, the MLP stand exhibited a negative total effect (β = −0.05) on bacterial function, suggesting that larch–spruce mixtures may suppress certain bacterial functional processes relative to a pure larch stand.

The pathways governing the fungal community were distinct from those observed for bacteria and were characterized by strong direct effects (Figure 8C). The MLP stand significantly shaped fungal community structure (β = 0.567, *p* < 0.05), in contrast to the MLB stand, which had a significant negative effect (β = −0.512, *p* < 0.05) (Figure 8D). In contrast to the bacterial community, neither leaf traits nor soil properties exerted significant direct influences on fungal community structure. The function of keystone fungal taxa was significantly and negatively affected by fungal community structure (β = −0.985, *p* < 0.01). Together, these results highlighted fundamental differences in the regulatory mechanisms of bacterial and fungal communities: bacterial composition appeared primarily influenced by leaf trait and soil-mediated pathways, whereas fungal communities responded more directly to tree species composition.

## 4. Discussion

### 4.1. Effects of Tree Species Mixing on Leaf Functional Traits and Soil Physicochemical Properties

In forest ecosystems, leaf morphological and physiological traits are closely linked to environmental conditions and resource availability [53,54]. Thus, increasing attention has been paid to the responses of key leaf functional traits to changes in stand environments. In the present study, several leaf traits differed significantly among the stand types, highlighting distinct aboveground strategies associated with different tree species mixtures. Specifically, LA, SLA, and LNC were significantly higher in the MLB stands than in the pure larch stands (Figure 2). These results are consistent with previous studies showing that mixed conifer–broadleaf forests typically promote larger leaf areas and higher specific leaf areas, thereby enhancing light interception and photosynthetic efficiency. The higher LNC in the MLB stands may result from greater light availability and faster growth rates, leading to increased nitrogen investment to meet higher metabolic demands [55,56]. However, MLP stands exhibited significantly higher LCC and LPC, suggesting a shift toward more carbon- and phosphorus-rich leaf tissues. These stands may experience greater competitive pressures, thereby promoting greater carbon and phosphorus allocation to stress-resistant tissues [57]. These contrasting leaf trait patterns indicate that the identity of admixed tree species, rather than tree species mixing per se, plays a critical role in shaping plant functional strategies.

Tree species mixing markedly altered soil properties, although the direction and magnitude of these changes differed between mixed stand types. MLB stands exhibited higher soil nutrient concentrations, particularly STN and STP, whereas MLP stands had significantly higher SOC levels but lower STN and SAN (Figure 2). Such shifts in soil nutrient availability were largely driven by variation in litter quality and decomposition dynamics among tree species [17]. In particular, needle litter from conifers generally contains a higher lignin content, exhibits a comparatively elevated C/N ratio, and is richer in phenolic compounds, leading to slower decomposition, delayed nutrient release, and consequently lower soil nutrient availability [58,59]. By comparison, stands composed of multiple tree species typically possess greater litter diversity, particularly those incorporating broadleaved species, which enhances nutrient turnover and improves soil fertility through complementary decomposition pathways [12,21]. However, STN and SAN levels in MLP stands were significantly reduced compared with those in pure larch plantations, likely due to the higher carbon content of the litter, which stimulates microbial activity and induces microbial immobilization of soil nitrogen [60]. This process can reduce the availability of nitrogen in the soil, which in turn alters nutrient dynamics in the stand. Collectively, these results indicate that while mixed stands can enhance soil nutrient status, the direction and magnitude of change are strongly determined by the functional traits and litter chemistry of the admixed tree species.

### 4.2. Effects of Tree Species Mixing on Soil Microbial Community Structure

The study reveals that mixed tree species exert both direct and indirect influences on soil microbial communities, with soil properties and leaf functional traits serving as pivotal mediators in structuring these communities. The MLB stand increased bacterial Chao1 richness, whereas the MLP stand reduced fungal alpha diversity (Figure 3). Moreover, stand type significantly structured both bacterial and fungal community composition, with both mixed larch stands clearly separated from the PL stand (Figure 4 and Table 1). This divergence is consistent with patterns documented in previous studies that tree species mixtures alter belowground microbial assemblages by modifying resource inputs and habitat conditions [12,61,62]. The envfit analysis further revealed that substantial heterogeneity in microbial communities was closely associated with soil physicochemical factors and leaf traits, including LCC, LPC, STN, and SAN. Variation partitioning indicated that differences in leaf functional traits together with soil conditions explained 16.90% and 17.46% of overall community variation, respectively. These moderate explanatory powers suggest that additional factors, including tree species identity, litter quality, and microbial interactions [63], also influence soil microbial assembly by providing more diverse nutritional substrates than those in the PL stand [64].

The PLS-PM results further demonstrated that mixed larch stands influenced bacterial and fungal communities through distinct pathways. We detected an absence of any direct pathway linking tree species mixing to bacterial community structure (Figure 8A). Nevertheless, tree species mixing indirectly affected bacterial communities through changes in leaf functional traits and soil properties. Although soil properties did not exhibit a statistically significant direct influence on bacterial community structure in our model, the considerable path coefficient (β = −0.387) indicates a potentially meaningful influence. The lack of statistical significance may result from the limited sample size rather than the absence of an effect, and future verification of this relationship will require studies employing larger datasets or more rigorously controlled experimental designs.

The two mixed-species plantations exerted contrasting total effects on bacterial community structure through tree species-mediated differences in specific leaf traits and soil properties (Figure 8B). The MLB stand generally displayed higher Leaf PC1 scores, reflecting a resource-acquisitive strategy characterized by greater SLA and LNC (Figure S1; Table S2). These traits are typically associated with higher nutrient availability and more labile litter [32,65]. The resulting accelerated litter decomposition promotes nutrient cycling and reduces sequestration [66], thus providing a richer and more readily assimilable energy source for specific bacterial taxa [65]. Consistent with this interpretation, Soil PC1, driven primarily by STN, STP, and SAN, was higher in MLB stands than in PL stands, reflecting enriched soil nutrient status, a key determinant of microbial diversity and community composition [66]. Across all stands, Actinobacteria, Proteobacteria, Acidobacteria, Verrucomicrobiota, and Chloroflexi represented a major fraction of the bacterial community (Figure 4C). Actinobacteria, known as copiotrophic decomposers of recalcitrant organic matter [67], and Verrucomicrobiota, which specialized in degrading complex carbohydrates [68,69], both exhibited lower relative abundances in the MLB stands compared with the PL stands, whereas Acidobacteria and Chloroflexi exhibited the opposite trend. This pattern suggests that improved litter quality and nutrient availability in the MLB stands favor bacterial guilds adapted to more readily decomposable substrates, thereby restructuring microbial niche through environmental filtering and competitive exclusion. In contrast, the MLP stand showed reduced Leaf PC1 scores, reflecting a resource-conservative strategy characterized by elevated LCC and LPC but lower SLA and LNC [70]. These traits are typically associated with the production of low-quality, chemically resistant litter [65], which, together with reduced Soil PC1 scores, indicates lower soil nitrogen and phosphorus availability. Under such conditions, bacterial communities may shift toward taxa adapted to nutrient-poor environments and slow growth rates. Consistent with this pattern, MLP stands showed higher relative abundances of Proteobacteria and Acidobacteria, but reduced Actinobacteriota, indicating a restructuring of below-ground bacterial communities toward low-nutrient-adapted and slow-growing taxa [16].

Fungal community structure, however, was predominantly shaped by the identity of the mixed tree species rather than environmental variables. Both PLS-PM and community composition analyses revealed a higher abundance of Basidiomycota and lower abundances of Mortierellomycota and Ascomycota in MLP stands relative to PL stands (Figure 4D). This supported earlier studies demonstrating that ectomycorrhizal fungi, particularly Basidiomycota, are selectively promoted by spruce and other coniferous hosts [71,72,73]. Furthermore, the MLB stand showed reduced Ascomycota abundance relative to the PL stand, which may reflect competitive exclusion of larch-associated endophytic fungi following the introduction of birch, which alters host availability and symbiotic compatibility [74]. Collectively, these findings support the view that tree identity, rather than the sheer level of species richness, primarily drives fungal community changes [17,64,75].

### 4.3. Effects of Tree Species Mixing on the Function of Keystone Soil Microbial Taxa

Our findings in larch-based mixed stands align well with those of Li et al. [76], who reported that species mixtures can alter dominant microbial taxa and their associated functions. Based on the functional characterization of keystone bacterial taxa, we found that mixed stands shaped bacterial community functions indirectly via their impact on soil properties, with the MLB stand exhibiting a pronounced positive effect (Figure 8A). These keystone taxa were closely correlated with essential soil nutrients, particularly STP and SAN (Figure 7A). Functionally, bacterial groups associated with chemoheterotrophy, nitrification, nitrate reduction, and respiration were significantly enriched in MLB stands (Figure 6A). Chemoheterotrophy is essential to ecosystem organic matter dynamics, as chemoheterotrophic microorganisms use organic carbon as both an energy and carbon source for cellular biosynthesis [77]. The enhanced abundance of these functional groups suggested that the MLB stand strengthened its organic matter decomposition capacity by increasing key soil nutrients, including nitrogen and phosphorus (high Soil PC1). The introduction of birch also modified the keystone bacterial taxa (Table S5), thereby diversifying nitrogen cycling pathways and fostering the development of a more complex microenvironment. Key taxa such as *Nitrospira*, a core nitrifier that acts as the main biological source of plant-available nitrate [78], and *Romboutsia*, which cooperates with other taxa to accelerate soil organic carbon decomposition and energy flow [76], collectively facilitated soil carbon and nitrogen cycling and contributed to improved soil fertility. These findings underscore the importance of keystone bacterial taxa as pivotal regulators of soil biogeochemical processes [79,80]. Conversely, the MLP stand had a negative total effect on bacterial ecological function (Figure 8D). In its bacterial community, the relative abundance of chemoheterotrophy—a key energy and metabolism process—was reduced, while the function of dark hydrogen oxidation was slightly enhanced. This pattern suggests that under relatively resource-limited conditions (low Soil PC1), the bacterial community in the MLP stand shifted toward alternative metabolic strategies. The dominant keystone taxa, such as *Mycobacterium*, KD4-96, and *Conexibacter*, which are typically adapted to oligotrophic, acidic, and carbon-limited environments, dominated the network [81,82]. These taxa are commonly associated with slow growth rates and conservative resource-use strategies, potentially constraining overall bacterial functional output under nutrient-poor conditions.

In contrast to bacteria, larch–birch and larch–spruce mixtures exerted no significant effect on the function of keystone fungal taxa via changes in leaf functional traits (Leaf PC1) or soil nutrient availability (Soil PC1). This suggested that fungal ecological functions were relatively insensitive to the observed divergence in leaf and soil traits between the two larch-based mixed stands. The weaker correlations between keystone fungal taxa and abiotic factors (Figure 7B) further support this interpretation. Notably, the PLS-PM revealed a strong direct negative effect of fungal community structure on its ecological function (Figure 8C), indicating that changes in network organization rather than resource availability primarily governed functional outcomes. Generally, community stability increases as network and modular structures become more complex [83]. The introduction of birch simplified the original fungal co-occurrence network (Figure 5B), potentially shifting the community toward a more unstable state. The MLB stand contained a higher number of keystone fungal taxa, predominantly belonging to *Cadophora* and *Exophiala* (Table S5), which, as saprotrophs and opportunistic pathogens, significantly facilitate the transformation of recalcitrant organic matter [84,85]. Consistently, functional predictions revealed that the MLB stands had the highest relative abundances of plant pathogens and fungal parasites (Figure 6D), which may collectively suppress overall fungal ecological function despite enhanced decomposition potential. In contrast, the MLP stand, characterized by increased Basidiomycota and decreased Ascomycota relative to the PL stand (Figure 4D), also exhibited an increased relative abundance of the ectomycorrhizal functional group (Figure 6D). As the principal phylum of ectomycorrhizal fungi across these boreal and temperate biomes [86], Basidiomycota facilitates host nutrient uptake (e.g., in spruce) through extensive mycelial networks while suppressing saprotrophic mineralization. Thus, the results highlight that the identity of admixed tree species serves as a central driver governing not only the structural assembly but also the functional expression of the soil fungal community.

## 5. Conclusions

This study examined plant functional traits, soil properties, and microbial communities in a pure larch plantation and two larch-based mixed stands to elucidate the mechanisms of belowground ecosystem differentiation in response to tree species mixing. A central finding was that larch–birch and larch–spruce mixtures regulate soil microbial communities through distinct pathways. Bacterial communities were primarily influenced indirectly by changes in leaf functional traits and soil nutrient status, whereas fungal communities were shaped primarily by tree species identity. Importantly, the identity of the admixed species led to divergent functional outcomes. The MLB stand created a nutrient-enriched soil environment dominated by bacteria, along with saprotrophic and pathogenic fungi, thereby promoting rapid nutrient turnover. In contrast, the MLP stand favored ectomycorrhizal fungal networks, associated with reduced short-term mineralization but potentially enhanced long-term carbon storage and ecosystem stability. These findings suggest that mixed plantation design should be tailored to specific management objectives: larch–birch mixtures may be preferable for improving soil fertility and nutrient cycling, whereas larch–spruce mixtures may be better suited for carbon sequestration and long-term ecosystem stability.

## Figures and Tables

**Table 1:** Results of PERMANOVA assessing the influence of selected predictors on bacterial and fungal assemblages using Bray–Curtis distance. Asterisks represent statistical significance (* *p* < 0.05; ** *p* < 0.01; *** *p* < 0.001).

Taxonomy	Dissimilarity Group	PERMANOVA		
		F	*R* ^2^	*p*
Bacteria	Stand type	1.549	0.205	0.001 ***
	MLB vs. PL	1.312	0.141	0.047 *
	MLP vs. PL	1.774	0.182	0.005 **
	MLP vs. MLB	1.551	0.162	0.012 *
Fungi	Stand type	1.545	0.205	0.001 ***
	MLB vs. PL	1.445	0.153	0.025 *
	MLP vs. PL	1.780	0.182	0.009 **
	MLP vs. MLB	1.431	0.152	0.014 *

**Table 2:** Leaf traits and soil variables used for vector fitting against the NMDS ordination of bacterial and fungal communities. Values were calculated using the “envfit” function with 999 permutations. Bold values indicate statistically significant at *p* < 0.05.

	Bacteria		Fungi	
	*R* ^2^	*p*-Value	*R* ^2^	*p*-Value
LA	0.27	0.154	0.05	0.751
SLA	0.18	0.290	**0.46**	**0.020**
LDMC	0.08	0.596	0.08	0.610
LCC	**0.51**	**0.025**	**0.48**	**0.019**
LNC	0.29	0.134	**0.60**	0.004
LPC	**0.43**	**0.027**	**0.74**	**0.005**
SOC	0.22	0.198	0.14	0.423
STN	**0.49**	**0.012**	**0.74**	**0.002**
STP	0.27	0.159	0.30	0.111
SAN	**0.58**	**0.008**	**0.48**	**0.018**
SAP	0.03	0.854	0.04	0.774
pH	0.06	0.671	0.23	0.202

## Data Availability

Data generated or analyzed during this study are available from the corresponding author upon reasonable request.

## Supplementary Materials

The following supporting information can be downloaded at https://www.mdpi.com/article/10.3390/biology15030259/s1, Figure S1. Principal component analysis (PCA) ordinations of leaf functional traits (A) and soil properties (B) across stand types and variance explained by principal axes (C); Figure S2. Compositional profiles of the dominant soil bacterial (A) and fungal (B) genera; Table S1. General information of sample plots; Table S2. Results of the principal component analysis (PCA) of 12 leaf functional traits and soil properties, showing the variable loadings, eigenvalues, and the proportion of variance explained by the first two principal components; Table S3. Best predictors of bacterial and fungal community structures across different stand types based on multiple linear regression; Table S4. Topological properties of bacterial and fungal networks in different stand types; Table S5. Keystone taxa in bacterial and fungal co-occurrence networks across different forest stand types.
