# Supplementary material for: Plant Functional Traits and Soil Properties Shape Soil Microbial Communities in Larix principis-rupprechtii Mixed Plantations
Source: Biology (Basel). 2026 Jan 30;15(3):259. doi: 10.3390/biology15030259 (PMC12896781; doi:10.3390/biology15030259)
Supplement: Supplementary file 1 [file biology-15-00259-s001.zip › biology-4116913-supplementary.pdf]

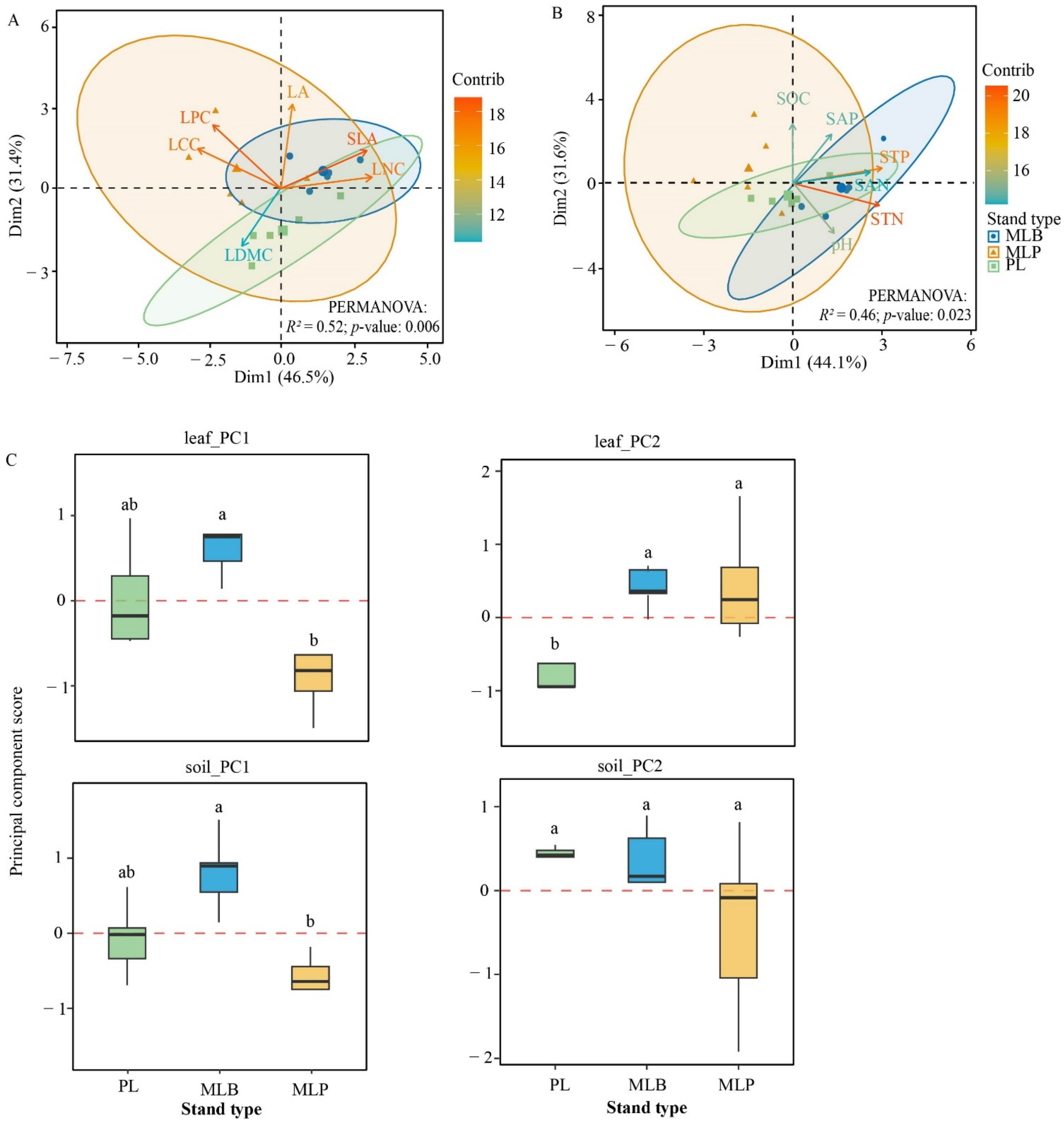

Figure S1. Principal component analyses (PCA) ordinations of leaf functional traits (A) and soil properties (B) across stand types and variance explained by principal axes (C). Different lowercase letters indicate significant differences at $p < 0.05$ according to Dunn's multiple comparison test following the Kruskal-Wallist test. PCA loadings can be found in Table S2.

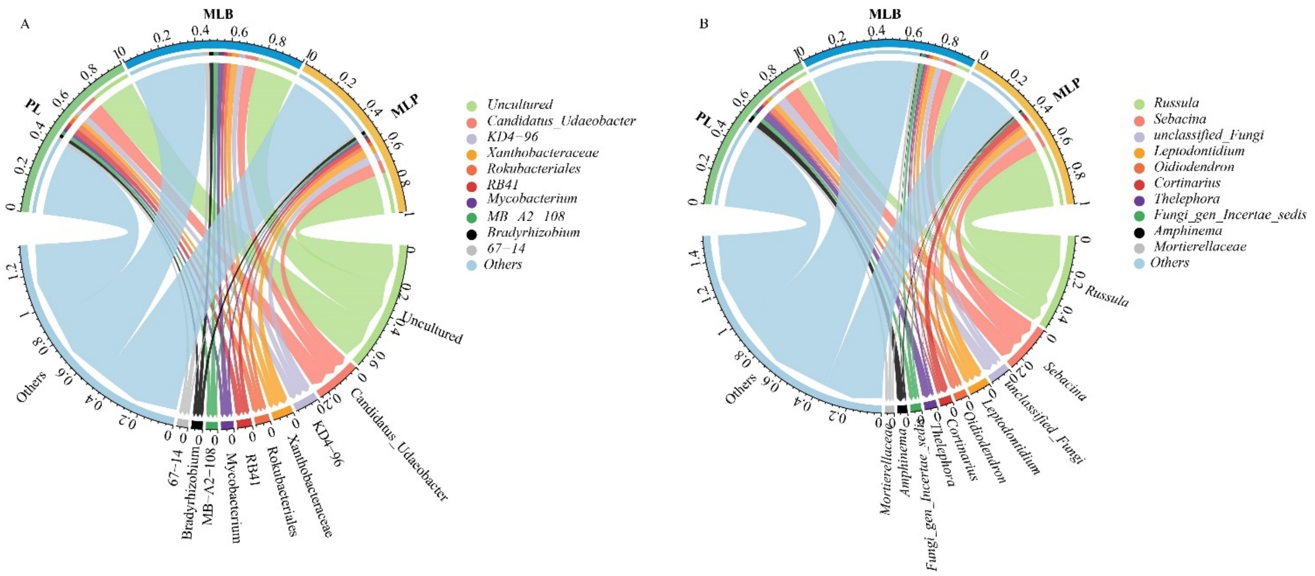

Figure S2. Compositional profiles of the dominant soil bacterial (A) and fungal (B) genera.

Table S1 General information of sample plots

Stand types	Stand ages (a)	Density (n tree hm^{-1})	Mixed ratio	Tree species	DBH (cm)	Height (m)	Average crown (m)
PL	42	1166±78	1:0	*Larix principis-rupprechtii*	17.92±2.12	14.96±2.57	3.24±0.32
MLB	44	1261±64	6:4	*Larix principis-rupprechtii*	20.36±1.41	16.42±2.71	3.49±0.72
				Betula platyphylla	16.98±2.16	14.24±1.80	3.64±0.85
MLP	48	1105±14	6:4	*Larix principis-rupprechtii*	26.09±5.96	18.42±4.55	3.94±1.36
				Picea asperata	23.86±6.76	16.47±5.74	3.58±1.08

Table S2 Results of the principal component analysis (PCA) of 12 leaf functional traits and soil properties, showing the variable loadings, eigenvalues, and the proportion of variance explained by the first two principal components.

	Leaf PC1	Leaf PC2		Soil PC1	Soil PC2
Eigenvalue	2.79	1.89	Eigenvalue	2.65	1.89
Proportion Explained (%)	46.5	31.4	Proportion Explained (%)	44.1	31.6
Cumulative Proportion (%)	46.5	77.9	Cumulative Proportion (%)	44.1	75.7
Loading scores			Loading scores		
LA	0.138	1.088	SOC	-0.002	-1.022
SLA	1.050	-0.725	STN	1.127	0.406
LDMC	-0.466	0.491	STP	1.138	-0.265
LCC	-1.006	0.509	SAN	0.978	-0.194
LNC	1.116	0.149	SAP	0.497	-0.879
LPC	-0.816	0.806	pH	0.522	0.896

Table S3. Best predictors of bacterial and fungal community structures across different stand types based on multiple linear regression. The table shows the coefficient of determination (R^2) for each model, along with the regression coefficients (Estimate) and *t*- value of the predictors. * Significance levels: $p < 0.05$.

	Bacterial community			Fungal community		
	$R^2 = 0.438$ *			$R^2 = 0.315$		
Predictors	Est.	*t*- value	*p*	Est.	*t*- value	*p*
Leaf PC1	-3.73	-1.09	0.297	-3.25	-2.22	0.047
Soil PC1	-3.58	-2.86	0.014	-3.09	-0.77	0.455

Table S4. Topological properties of bacterial and fungal networks in different stand types

Network metrics	Bacterial network			Fungal network		
	PL	MLB	MLP	PL	MLB	MLP
Number of nodes	330	575	441	240	79	72

Number of edges	1369	4210	2535	1017	168	120
Modularity	0.861	0.827	0.814	0.812	0.795	0.766
Network diameter	18	20	21	27	8	15
Average path length	5.687	8.197	8.256	8.951	2.827	5.739
Average degree	8.297	14.643	11.497	8.475	4.253	3.333
Average clustering coefficient	0.807	0.758	0.757	0.768	0.819	0.671
Graph density	0.025	0.026	0.026	0.035	0.055	0.047

Table S5. Keystone taxa in bacterial and fungal co-occurrence networks across different stand types.

Stand types	ID	Network roles	Taxonomy	Phylum	Genus	Total abundance
PL	ASV_5013	module hubs	Bacteria	Proteobacteria	*Mesorhizobium*	21
	ASV_76110	module hubs	Bacteria	Proteobacteria	*uncultured*	143
	ASV_125557	module hubs	Bacteria	Verrucomicrobiota	*Candidatus_Udaeobacter*	4
	ASV_149244	module hubs	Bacteria	Actinobacteriota	*Kineosporia*	16
	ASV_168302	module hubs	Bacteria	Chloroflexi	*KD4-96*	8
	ASV_198851	module hubs	Bacteria	Acidobacteriota	*uncultured*	47
	ASV_242762	module hubs	Bacteria	Proteobacteria	*TRA3-20*	24
	ASV_433822	module hubs	Bacteria	Actinobacteriota	*uncultured*	9
	ASV_447601	module hubs	Bacteria	Gemmatimonadota	*uncultured*	11
	ASV_163670	connectors	Bacteria	Proteobacteria	*Bradyrhizobium*	19
	ASV_194748	connectors	Bacteria	Verrucomicrobiota	*Candidatus_Udaeobacter*	3
	ASV_204815	connectors	Bacteria	Verrucomicrobiota	*Candidatus_Udaeobacter*	25
	ASV_404981	connectors	Bacteria	Acidobacteriota	*uncultured*	10
	ASV_1748	module hubs	Fungi	Fungi_phy_Incertae_sedis	*Fungi_gen_Incertae_sedis*	6
	ASV_19845	module hubs	Fungi	Ascomycota	*Beauveria*	11
	ASV_20643	module hubs	Fungi	Mortierellomycota	*Mortierella*	110
	ASV_21549	module hubs	Fungi	Ascomycota	*unclassified_Coniochaetaceae*	7
	ASV_23739	module hubs	Fungi	Mortierellomycota	*Linnemannia*	14

	ASV_28133	module hubs	Fungi	Ascomycota	*Hypocreaceae_gen_Incertae_sedis*	17
	ASV_28409	module hubs	Fungi	Ascomycota	*unclassified_Hypocreales*	9
	ASV_31727	module hubs	Fungi	Ascomycota	*Lasionectriopsis*	10
	ASV_35335	module hubs	Fungi	Mortierellomycota	*Linnemannia*	242
	ASV_39048	module hubs	Fungi	Basidiomycota	*Hygrophorus*	455
	ASV_43544	module hubs	Fungi	Ascomycota	*Pseudogymnoascus*	993
	ASV_7482	connectors	Fungi	Basidiomycota	*Trechispora*	355
	ASV_19703	connectors	Fungi	Ascomycota	*Lophium*	4
	ASV_41701	connectors	Fungi	Fungi_phy_Incertae_sedis	*Fungi_gen_Incertae_sedis*	1
	ASV_42535	connectors	Fungi	Mortierellomycota	*Dissophora*	44
	ASV_44291	connectors	Fungi	Ascomycota	*Thermomyces*	2
	ASV_44463	connectors	Fungi	Ascomycota	*Leohumicola*	3
	ASV_44818	connectors	Fungi	Ascomycota	*Fusicolla*	22
	ASV_48011	connectors	Fungi	Ascomycota	*unclassified_Sordariales*	2
	ASV_54620	connectors	Fungi	Fungi_phy_Incertae_sedis	*Fungi_gen_Incertae_sedis*	2
MLB	ASV_10212	module hubs	Bacteria	Firmicutes	*Romboutsia*	2
	ASV_41782	module hubs	Bacteria	Proteobacteria	*uncultured*	7
	ASV_44986	module hubs	Bacteria	Proteobacteria	*Rhodoplanes*	1
	ASV_71594	module hubs	Bacteria	Acidobacteriota	*uncultured*	8
	ASV_81052	module hubs	Bacteria	Chloroflexi	*Gitt-GS-136*	5
	ASV_86053	module hubs	Bacteria	Acidobacteriota	*Subgroup_2*	4
	ASV_96584	module hubs	Bacteria	Verrucomicrobiota	*Candidatus_Udaeobacter*	4
	ASV_171372	module hubs	Bacteria	Proteobacteria	*uncultured*	7
	ASV_207134	module hubs	Bacteria	Actinobacteriota	*Gaiella*	1
	ASV_237303	module hubs	Bacteria	Proteobacteria	*unclassified_Xanthobacteraceae*	5
	ASV_238886	module hubs	Bacteria	Chloroflexi	*uncultured*	2

	ASV_258162	module hubs	Bacteria	Nitrospirota	*Nitrospira*	18
	ASV_264375	module hubs	Bacteria	Bacteroidota	*Mucilaginibacter*	2
	ASV_266412	module hubs	Bacteria	Chloroflexi	*TK10*	5
	ASV_287479	module hubs	Bacteria	Actinobacteriota	*Conexibacter*	2
	ASV_305326	module hubs	Bacteria	Proteobacteria	*Bradyrhizobium*	4
	ASV_349647	module hubs	Bacteria	Acidobacteriota	*RB41*	1
	ASV_420298	module hubs	Bacteria	Acidobacteriota	*Subgroup_5*	5
	ASV_423377	module hubs	Bacteria	Proteobacteria	*uncultured*	2
	ASV_201106	connectors	Bacteria	Proteobacteria	*uncultured*	26
	ASV_248784	connectors	Bacteria	Chloroflexi	*KD4-96*	19
	ASV_286001	connectors	Bacteria	Chloroflexi	*KD4-96*	25
	ASV_387047	connectors	Bacteria	Acidobacteriota	*uncultured*	11
	ASV_2665	module hubs	Fungi	Ascomycota	*unclassified_Pseudeurotiaceae*	23
	ASV_3616	network hubs	Fungi	Chytridiomycota	*Lobulomycetales_gen_Incertae_sedis*	3
	ASV_15533	module hubs	Fungi	Ascomycota	*Cadophora*	8
	ASV_36563	module hubs	Fungi	Ascomycota	*Exophiala*	46
	ASV_37798	connectors	Fungi	unclassified_Fungi	*unclassified_Fungi*	10
MLP	ASV_234953	module hubs	Bacteria	Acidobacteriota	*uncultured*	1
	ASV_313315	module hubs	Bacteria	Acidobacteriota	*uncultured*	1
	ASV_370514	module hubs	Bacteria	Acidobacteriota	*Subgroup_17*	6
	ASV_37306	connectors	Bacteria	Myxococcota	*Haliangium*	8
	ASV_60385	connectors	Bacteria	Chloroflexi	*TK10*	16
	ASV_69824	connectors	Bacteria	Actinobacteriota	*Mycobacterium*	4
	ASV_78975	connectors	Bacteria	Actinobacteriota	*Mycobacterium*	1
	ASV_102085	connectors	Bacteria	Proteobacteria	*unclassified_Xanthobacteraceae*	16
	ASV_108493	connectors	Bacteria	Proteobacteria	*unclassified_Xanthobacteraceae*	7
	ASV_130272	connectors	Bacteria	Proteobacteria	*uncultured*	3

ASV_130846	connectors	Bacteria	Verrucomicrobiota	*Candidatus_Udaeobacter*	23
ASV_133626	connectors	Bacteria	Actinobacteriota	*Solirubrobacter*	2
ASV_139934	connectors	Bacteria	Actinobacteriota	*Pseudonocardia*	5
ASV_162112	connectors	Bacteria	Acidobacteriota	*uncultured*	1
ASV_168049	connectors	Bacteria	Actinobacteriota	*Mycobacterium*	8
ASV_229764	connectors	Bacteria	Acidobacteriota	*uncultured*	5
ASV_268623	connectors	Bacteria	Actinobacteriota	*Streptomyces*	20
ASV_280001	connectors	Bacteria	Acidobacteriota	*RB41*	10
ASV_284090	connectors	Bacteria	Myxococcota	*bacteriap25*	82
ASV_313488	connectors	Bacteria	Acidobacteriota	*Subgroup_17*	4
ASV_324041	connectors	Bacteria	Actinobacteriota	*Conexibacter*	15
ASV_353962	connectors	Bacteria	Actinobacteriota	*Mycobacterium*	8
ASV_405303	connectors	Bacteria	Desulfobacterota	*Geobacter*	27
ASV_405581	connectors	Bacteria	Chloroflexi	*KD4-96*	1
ASV_480177	connectors	Bacteria	Actinobacteriota	*uncultured*	5
ASV_13515	module hubs	Fungi	unclassified_Fungi	*unclassified_Fungi*	2
ASV_15533	module hubs	Fungi	Ascomycota	*Cadophora*	12
ASV_19729	module hubs	Fungi	Ascomycota	*unclassified_Mycosphaerellaceae*	33
ASV_40912	module hubs	Fungi	Ascomycota	*Pleotrichocladium*	234
ASV_44413	module hubs	Fungi	Mortierellomycota	*Podila*	249
ASV_3233	connectors	Fungi	Mortierellomycota	*Linnemannia*	29
ASV_12234	connectors	Fungi	Ascomycota	*Setophoma*	291
